# To Refer or Defer: Closing the Referral Gap in Severe Tricuspid Regurgitation

**DOI:** 10.1016/j.shj.2026.101115

**Published:** 2026-09-23

**Authors:** Benjamin Titus, Firas Zahr

**Affiliations:** Division of Cardiovascular Medicine, Knight Cardiovascular Institute, Oregon Health and Science University, Portland, Oregon, USA

Severe tricuspid regurgitation (TR) was once dismissed as an incidental finding and managed conservatively. It is now recognized as an independent predictor of morbidity and mortality, with prognosis similar to that of left-sided valve disease.^1^ Historically, definitive treatment options, such as isolated surgical repair, carried substantial operative risk. Recent advancements in transcatheter therapies now offer select patients a nonsurgical option, such as tricuspid transcatheter edge-to-edge repair (T-TEER) or transcatheter tricuspid valve replacement (TTVR). The pivotal trials for these devices have yet to demonstrate a mortality benefit, but they have shown a meaningful improvement in symptoms and quality of life.^2^^,^^3^ Despite Food and Drug Administration approval and updated guidelines recommending heart team evaluation, referrals and procedural volume have remained low.^4^, ^5^, ^6^ The question has shifted from whether we can treat severe TR to whether patients are being referred early enough to benefit from specialist evaluation.

In this issue of *Structural Heart*, Darko et al^7^ help clarify this referral problem. They analyzed referral patterns for 1000 patients with severe TR in a single-center registry and found that only 32.8% were evaluated by a structural heart disease specialist. This was a high-risk cohort: 86% had heart failure, 65% had kidney disease, 33% had liver disease, and symptoms were present in the vast majority. Referral tracked most closely with a heart failure diagnosis (adjusted odds ratio for nonevaluation, 0.20), New York Heart Association class III–IV symptoms, coronary artery disease, and conduction delay. Among patients without a heart failure diagnosis, only 13.2% were evaluated. This suggests that clinicians rely most heavily on heart failure as a marker of disease severity, as other markers such as kidney disease and liver disease carried no independent weight. Right ventricular systolic pressure predicted both a lower likelihood of referral and worse survival. Referred patients initially appeared to have better survival, but this association was attenuated after adjustment for referral bias and immortal time bias. The authors interpret this finding cautiously, as the pivotal trials discussed previously failed to show any mortality benefit.

The central challenge is distinguishing missed referral opportunities from appropriate triage. Doing so requires careful assessment of disease stage, including whether symptoms reflect progressive valve disease or advanced right-sided heart failure. It also depends on underlying TR etiology and echocardiographic parameters such as right ventricular systolic pressure, right ventricular function, coaptation gap, leaflet anatomy, and interaction with device leads, each of which may affect candidacy for T-TEER or TTVR. These features must be interpreted in the context of a patient’s unique hemodynamic state at the time of evaluation, because optimization with diuresis or other “prehabilitation” strategies may improve procedural eligibility for those initially deemed unsuitable for intervention.^8^ Without a more complete understanding of each patient’s clinical context, it is difficult to connect evaluation and treatment decisions to observed clinical outcomes.

Despite its limitations, the article offers valuable insight into a single center’s experience in treating tricuspid valve disease. This is a multifaceted problem that depends on identifying appropriate patients, ensuring access to care, and addressing provider bias. Across the transcatheter literature, patients with severe TR are frequently referred only after irreversible right heart failure has developed, at which point intervention carries greater risk and outcomes are worse.^9^ The problem is compounded by the small number of centers offering T-TEER or TTVR, which forces patients in underserved areas to travel farther to reach an eligible hospital.^5^ Finally, uncertainty persists about the true clinical benefit of transcatheter tricuspid therapies due in part to older surgical data showing poor outcomes after isolated tricuspid valve surgery.^10^ Others remain cautiously optimistic citing improvements in symptoms, right ventricular remodeling, and possible gains in renal and hepatic function.^2^^,^^3^^,^^11^

For these reasons, attributing a low referral rate to under-recognition alone may be an oversimplification. In some cases, the choice to defer may have reflected appropriate clinical judgment rather than a missed opportunity. This distinction helps explain why standardized referral pathways used in other valve diseases may not translate directly to severe TR. Clinicians may eventually leverage machine learning processes to incorporate echocardiographic, clinical, and laboratory data into the referral process, rather than relying on a single International Classification of Diseases-10 diagnosis code. Early evidence from the cluster-randomized ALERT trial suggests that machine learning algorithms may improve management of significant aortic stenosis or mitral regurgitation, resulting in higher rates of valve intervention or multidisciplinary heart team evaluation within 90 days (win ratio, 1.27; *p* = 0.007) when compared to usual care.^12^ Whether a similar artificial intelligence–driven detection-and-alert strategy could be adapted to tricuspid disease remains untested, but the aortic stenosis/mitral regurgitation experience suggests that earlier, algorithm-assisted referral is achievable and worth pursuing. Darko et al have taken a valuable first step in defining the problem and proposing a possible solution. The next challenge will be closing the gap between referral and deferral and determining whether timely referral translates into meaningful improvement of patient outcomes.

## Funding

The authors have no funding to report.

## Disclosure Statement

Firas Zahr receives research and educational grants and is consultant for Edwards Lifesciences, Philips, GE healthcare and Medtronic.

The other authors had no conflicts to declare.
